# Parenchymal preserving anatomic resections result in less pulmonary function loss in patients with Stage I non-small cell lung cancer

**DOI:** 10.1186/s13019-015-0253-6

**Published:** 2015-04-01

**Authors:** Ryan A Macke, Matthew J Schuchert, David D Odell, David O Wilson, James D Luketich, Rodney J Landreneau

**Affiliations:** 1 Division of Cardiothoracic Surgery; Department of Surgery, University of Wisconsin, 600 Highland Avenue, H4/318 Clinical Sciences Center, Madison, WI 53792 USA; 2 Division of Thoracic and Foregut Surgery; Department of Cardiothoracic Surgery, University of Pittsburgh, Pittsburgh, PA USA; 3 Division of Cardiothoracic Surgery, Department of Surgery; Alleghany Health Network, Pittsburgh, PA USA

**Keywords:** Pulmonary function, Lung segmentectomy or wedge resection, Lobectomy (lung), Lung cancer, Outcomes

## Abstract

**Background:**

A suggested benefit of sublobar resection for stage I non-small cell lung cancer (NSCLC) compared to lobectomy is a relative preservation of pulmonary function. Very little objective data exist, however, supporting this supposition. We sought to evaluate the relative impact of both anatomic segmental and lobar resection on pulmonary function in patients with resected clinical stage I NSCLC.

**Methods:**

The records of 159 disease-free patients who underwent anatomic segmentectomy (n = 89) and lobectomy (n = 70) for the treatment of stage I NSCLC with pre- and postoperative pulmonary function tests performed between 6 to 36 months after resection were retrospectively reviewed. Changes in forced expiratory volume in one second (FEV_1_) and diffusion capacity of carbon monoxide (DLCO) were analyzed based upon the number of anatomic pulmonary segments removed: 1–2 segments (n = 77) or 3–5 segments (n = 82).

**Results:**

Preoperative pulmonary function was worse in the lesser resection cohort (1–2 segments) compared to the greater resection group (3–5 segments) (FEV_1(%predicted)_: 79% vs. 85%, p = 0.038; DLCO_(%predicted)_: 63% vs. 73%, *p* = 0.010). A greater decline in FEV_1_ was noted in patients undergoing resection of 3–5 segments (FEV_1 (observed)_: 0.1 L vs. 0.3 L, *p* = 0.003; and FEV_1 (% predicted)_: 4.3% vs. 8.2%, *p* = 0.055). Changes in DLCO followed this same trend (DLCO_(observed)_: 1.3 vs. 2.4 mL/min/mmHg, *p* = 0.015; and DLCO_(% predicted)_: 3.6% vs. 5.9%, *p* = 0.280).

**Conclusions:**

Parenchymal-sparing resections resulted in better preservation of pulmonary function at a median of one year, suggesting a long-term functional benefit with small anatomic segmental resections (1–2 segments). Prospective studies to evaluate measurable functional changes, as well as quality of life, between segmentectomy and lobectomy with a larger patient cohort appear justified.

## Background

Arguments over the extent of parenchymal resection for the small peripheral lung cancer have been waged since the earliest attempts at surgical management of this disease [1-9]. The primary concern of oncologic adequacy of resection between sublobar resection and lobectomy or pneumonectomy has always been the primary point of contention. However, preservation of vital pulmonary function has also been a major consideration in choosing less than lobectomy for the small peripheral lung cancer. There are conflicting reports in the surgical literature related to the utility of sublobar resection, particularly anatomic segmentectomy, in preserving precious lung function compared to lobectomy for early stage lung cancer [8,10-13]. These conflicting reported outcomes led us to retrospectively review our experience with anatomic segmentectomy and lobectomy for disease-free, stage I peripheral NSCLC patients to assess the long-term impact of resection on pulmonary function.

## Methods

This study was approved by the Institutional Review Board of the University of Pittsburgh. The need for individual consent was waived given the retrospective nature of the analysis. Disease-free patients with a history of clinical stage I NSCLC who underwent surgical resection by anatomic segmentectomy (n = 89) or lobectomy (n = 70) from 2002 to 2010 with full preoperative and postoperative pulmonary function testing (PFT) were identified from the Thoracic Tumor Registry and billing records of the Department of Cardiothoracic Surgery at the University of Pittsburgh Medical Center. Only patients with PFT’s performed between 6 months to 36 months postoperatively were included in this study. Data collected from electronic or paper medical records included patient demographics, PFT results, operative details, and tumor characteristics (Table 1).

**Table 1:** **Preoperative demographics, tumor characteristics, and operative details**

	1-2 Segments	3-5 Segments	*P*value
	(n = 77)	(n = 82)	
Male sex	33 (42.9%)	42 (51.2%)	0.341
Age	69 ± 9	69 ± 10	0.818
Tumor size	1.9 ± 0.9	2.9 ± 1.9	<0.001
Tumor location			
Right upper lobe	27 (35.1%)	28 (34.1%)	1
Right middle lobe	9 (11.7%)		
Right lower lobe	9 (11.7%)	20 (24.4%)	0.042
Left upper lobe	27 (35.1%)	12 (14.6%)	0.003
Left lower lobe	5 (6.5%)	22 (26.8%)	<0.001
Approach			0.257
Thoracotomy	27 (35.1%)	37 (45.1%)	
VATS	50 (64.9%)	45 (54.9%)	
Mean # of segments resected	1.4 ± 0.5	4.0 ± 0.8	<0.001

As was the methodology of an earlier report assessing pulmonary functional loss differences between segmentectomy and lobectomy done by Harada et al. [11], each lobe of the lungs was segregated into their classical anatomic segments. The lower lobes were deemed to have 5 pulmonary segments, the right upper lobe to have 3 segments, the right middle lobe to have 2 segments, and left upper lobe to have 4 segments. Given that the basilar segmental group has 4 segments, the volume of lung parenchyma resected during basilar segmentectomy is more similar to that of an upper or lower lobectomy. Also, given that right middle lobes have only 2 segments, the volume resected for middle lobes is more consistent with a 1 or 2 segment segmentectomy. Therefore we chose to define the cohorts as those who had 1–2 segments resected (n = 77) and those who had 3–5 segments resected (n = 82). Basilar segmental resections were grouped in the greater resection cohort and middle lobectomies in the lesser resection cohort.

All patients underwent thorough preoperative staging with computed tomography scanning, with or without positron emission tomography, typically within 6 weeks of surgical resection. Additional diagnostic testing (brain magnetic resonance imaging, bone scan) was performed as warranted by patient symptoms and other clinical findings or at the discretion of the individual surgeon. Invasive mediastinal staging, such as endobronchial ultrasound with fine-needle aspiration or mediastinoscopy, was not routinely performed in the preoperative evaluation of these patients, unless mediastinal nodes were greater than 1 centimeter in diameter. All patients were clinically staged preoperatively as IA or IB according to the 7th edition of the tumor-node-metastases (TNM) classification of the American Joint Committee for Cancer Staging and the Revised International System for staging lung cancer [14].

The decision to proceed with segmentectomy or lobectomy was based primarily on surgeon preference for the management of stage I NSCLC; however, preoperative cardiopulmonary reserve, preoperative imaging, and comorbidities also influenced procedure selection on a patient-by-patient basis. Operative reports were reviewed and patients were excluded from analysis if the anatomic resection was not associated with identification, isolation, and individual division of the segmental broncho-vascular structures (i.e. extended wedge resection). The surgical approach was left to the discretion of the surgeon and included various open thoracotomy incisions or VATS (Table 1). Mediastinal, hilar, and interlobar lymph node sampling or dissection was routinely performed.

A complete evaluation of pulmonary function was carried out in all included patients preoperatively and at least 6 months following surgical resection, but no later than 36 months. The indication for obtaining interval PFT’s was variable, however the majority were obtained as part of a routine postoperative protocol for patients undergoing segmentectomy at our institution. Evaluation of pulmonary function was performed according to the standards outlined by the American Thoracic Society [15]. Forced expiratory volume in 1 second (FEV_1_) and diffusion capacity of carbon monoxide (DLCO) were measured in all patients pre- and postoperatively and were used for comparison between the types of surgical resection (1–2 segments vs. 3–5 segments). Given FEV_1_ and DLCO are the best-known spirometric predictors of postoperative morbidity and mortality, other spirometric values were not included in our analysis [16]. In cases where multiple measurements were reported for each value (i.e. pre- and post- bronchodilator treatment), the greatest measurement was used for the analysis. Observed measurements and percentage of standard values based on age, sex, height, and weight were included for analysis. The primary outcome in our study was the absolute and percent change from the preoperative observed and percent standard values for FEV_1_ and DLCO, which will be referred to as FEV_1 (observed)_, FEV_1 (% predicted)_, DLCO _(observed)_, and DLCO _(% predicted)_ in the remainder of the text.

Categorical variables (sex, approach, and tumor location) are reported as frequency and percentage with the Fischer’s exact test being used for comparative analysis. Continuous variables (age, tumor size, time of follow-up, and PFT results) are reported as mean with standard deviation or median with range, as appropriate. Comparative analysis of PFT results, including preoperative and postoperative values, as well as absolute and percent change was performed using the Mann–Whitney *U* test. Comparative analysis of preoperative and postoperative PFT results for each cohort was performed using the paired Student *t*-test. All comparisons were two-tailed. A *p-*value of 0.05 or less was considered statistically significant. The statistical package STATA, version 11.2 (College Station, TX) was used for the analyses.

## Results

Median follow-up, determined as the time of resection to the time of postoperative pulmonary function testing, was approximately 1 year for both groups (1–2 segments: 11.8 months, range 6.0 to 27.0; 3–5 segments 11.9 months, range 6.0 to 35.3; *p* = 0.952). Preoperative demographics, tumor characteristics, and operative details are shown in Table 1. No significant differences were noted in age or sex distribution when comparing the two cohorts. Patients who had 3–5 segments resected tended to have larger tumors (2.9 ± 1.9 centimeters) compared to those who had 1–2 segments resected (1.9 ± 0.9 centimeters, *p* = <0.001). Nearly three times as many segments were resected in those who underwent resection of 3–5 segments (4.0 ± 0.8 vs. 1.4 ± 0.5, *p =* <0.001). Fewer patients were resected via thoracotomy in the lesser resection cohort, though this difference was not statistically significant (35.1% vs. 45.1%, *p* = 0.257). Left upper lobe tumors were treated more frequently with resection of 1–2 segments (i.e. upper division segmentectomy or lingulectomy), whereas 3–5 segment resections were more commonly used to treat lower lobe tumors (Table 1). The distribution of segments and lobes resected is noted in Table 2.

**Table 2:** **Distribution by segments resected**

Type of resection	N = 159
3-5 segments	82
Right upper lobe	28
Right lower lobe	12
Right basilar segments	8
Left upper lobe	12
Left lower lobe	13
Left basilar segments	9
1-2 segments	77
Right apical segment	11
Right anterior segment	4
Right posterior segment	9
Right apicoposterior segments	3
Right middle lobe	5
Right medial segment	2
Right lateral segment	2
Right superior segment	9
Left upper division segments	17
Left lingular segments	4
Left anterior segment	1
Left apicoposterior segment	5
Left superior segment	5

**Figure 1:**
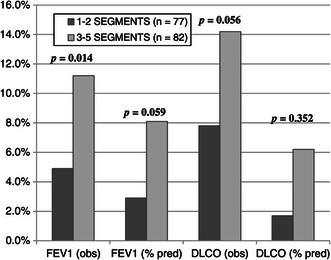
**Mean percent decline in PFT’s.**

PFT results for each cohort are detailed in Table 3. Patients undergoing resection of fewer anatomic segments had worse overall baseline pulmonary function prior to resection in regards to both FEV_1_ and DLCO. Parenchymal-sparing resections (1–2 segments) resulted in better preservation of postoperative pulmonary function compared to larger resections (3–5 segments) as demonstrated by the absolute decline in FEV_1 (observed)_ (107 mL vs. 286 mL; *p* = 0.003), FEV_1 (%predicted)_ (4.3% vs. 8.2%; *p* = 0.055), and DLCO _(observed)_ (1.5 vs. 2.6 mL/min/mmHg; *p* = 0.015). However, the decline in DLCO _(%predicted)_ (3.6% vs. 5.9%; *p* = 0.280) was not significantly different between resection groups. The mean percent decline in FEV_1_ and DLCO for patients undergoing resection of 1–2 and 3–5 segments is shown graphically in Figure 1.

**Table 3:** **Preoperative, postoperative, and mean decline in PFT’s**

	1-2 Segments	3-5 Segments	*P*value
	(n = 77)	(n = 82)	
Preoperative PFT’s			
FEV_1 (observed)_ (L)	1.95 ± 0.7	2.15 ± 0.7	0.085
FEV_1 (% predicted)_	79% ± 23	85% ± 21	0.038
D_LCO (observed)_	14.6 ± 5.4	16.6 ± 5.0	0.056
D_LCO (% predicted)_	63% ± 22	73% ± 19	0.010
Postoperative PFT’S			
FEV_1 (observed)_ (L)	1.85 ± 0.7	1.86 ± 0.6	0.726
FEV_1 (% predicted)_	75% ± 22	77% ± 21	0.631
D_LCO (observed)_	13.3 ± 5.8	14.2 ± 5.3	0.298
D_LCO (% predicted)_	60% ± 21	67% ± 22	0.060
Decline in PFT’s			
FEV_1 (obs)_ (L)	0.1 ± 0.3	0.3 ± 0.4	0.003
FEV_1 (% predicted)_	4.3% ± 17.4	8.2% ± 16.7	0.055
D_LCO (obs)_ (mL/min/mmHg)	1.3 ± 3.5	2.4 ± 3.6	0.015
D_LCO (% predicted)_	3.6% ± 15.8	5.9% ± 20.1	0.280

**Figure 2:**
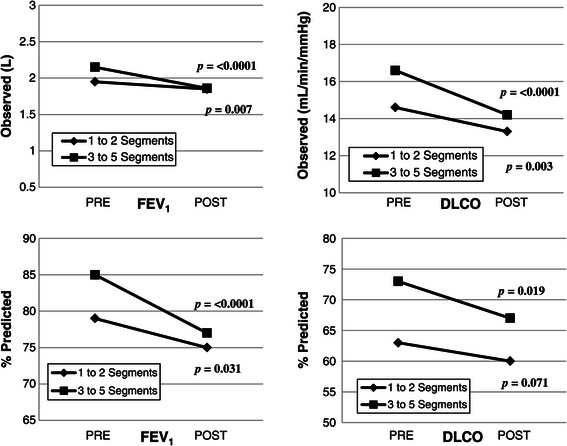
**Changes in pulmonary function based on paired analysis within each resection cohort.**

The results of the paired analysis comparing preoperative and postoperative values within each resection cohort are represented in Figure 2. The decline in DLCO _(%predicted)_ was not statistically significant following resection of 1–2 segments, whereas all other spirometric values analyzed declined significantly in both resection cohorts.

## Discussion

Beyond the arguments related to the oncologic adequacy of sublobar resection for the peripheral stage I lung cancer [17-19], a key component of determining the role of anatomic segmentectomy in the treatment of early-stage NSCLC involves evaluation of the theoretical advantage of better preservation of pulmonary function compared to lobectomy. To date, this question has received much less attention than the issues of morbidity, mortality, disease-free survival and overall survival. In addition, the few efforts focusing on this issue have yielded contradicting and controversial results [8,10-13]. Small patient populations, as well as variability in baseline pulmonary function and specific pulmonary function values reported in these studies have made it difficult to definitively validate this theory. To our knowledge, the current study represents the largest cohort of patients undergoing segmentectomy with pre- and postoperative pulmonary function testing available for comparison with lobectomy.

In the last two decades, there have been two studies comparing sublobar to lobar resection that have concluded that lesser resection does not provide an advantage in preservation of pulmonary function. The Lung Cancer Study Group reported statistically significant preservation of forced vital capacity (FVC) and FEV_1_ at 6 months postoperatively in favor of sublobar resection, however, after 12–18 months the benefit in FVC was no longer noted [8]. Interestingly, the authors stated that there was no difference in postoperative function between sublobar resection and lobectomy despite the percent difference of FEV_1_ remaining significant at 12–18 months (−5.2% vs. -11.1%; p = 0.041). Takizawa and colleagues compared anatomic segmentectomy to lobectomy for the treatment of small, peripheral NSCLC in a non-randomized, propensity-matched analysis [10]. They reported no difference in the change of FVC pre- and postoperatively at one year. However, a small but significant preservation in FEV_1_ following segmentectomy compared to lobectomy was noted.

In contrast, there have been three comparative studies that have provided data in support of better preservation in pulmonary function following segmentectomy. Harada and colleagues reported a significant benefit in preservation of FEV_1_ (*p* = 0.0007) and FVC (*p* = 0.0006) at six months following segmentectomy compared to lobectomy in patients with good baseline pulmonary function and stage I NSCLC’s less than 2 centimeters [11]. This study also demonstrated a significant correlation between number of segments resected and decrease in postoperative FEV_1_ (*p* = <0.0001) and FVC (*p* = <0.0001). Keenan and coworkers compared segmentectomy and lobectomy in the treatment of stage I NSCLC and found a significant decrease in FEV_1_, DLCO, and FVC at one year following lobectomy, whereas only DLCO declined significantly after anatomic segmentectomy [12]. Unlike the study by Harada et al., baseline FEV_1_ and DLCO were significantly worse in the segmentectomy cohort. Lastly, Martin-Ucar and colleagues compared changes in pulmonary function in a matched analysis of high-risk patients (predicted postoperative FEV_1_ < 40%) with stage I NSCLC treated with segmentectomy or lobectomy [13]. An increase in FEV_1_ was actually noted following segmentectomy in this study, in contrast to a significant decrease following lobectomy (12% vs. -12%; *p* = 0.02).

We took a slightly different approach in the present study. Since the purpose of our study was to determine if parenchymal sparing resections resulted in better preservation of pulmonary function, we chose to group patients by number of segments resected rather than simply comparing segmentectomies and lobectomies. Basilar segments are comprised of 4 pulmonary segments with the volume of lung resected more closely approximating that of a lobectomy, with the exception of 2 segment middle lobectomies. Basilar segmentectomies comprised 19% of segmentectomies in this series (17/89). In a separate analysis not included here, we found that when we compared all segmentectomies to lobectomies there was a trend toward preserved pulmonary function in the segmentectomy cohort, although none of these differences reached statistical significance. We suspect that the large proportion of basilar segmentectomies skewed these results given the greater volume of lung parenchyma resected relative to other segmentectomies. By grouping patients into 1–2 segment and 3–5 segment resections, more homogeneous cohorts were created based on volume of parenchyma resected.

Similar to the previously mentioned studies [11-13], we found that lesser resection resulted in better preservation of postoperative FEV_1_ at a median follow up time of 1 year. The question then becomes, is this difference clinically significant? Several Japanese authors have stated that a loss 200 mL or greater in FEV_1_ may be important when considering sublobar resection or lobectomy for patients with marginal pulmonary reserve [20,21]. Therefore, the mean decline in FEV_1(observed)_ of 107 mL seen following segmental resection of 1–2 segments is less likely to be clinically significant for most patients, whereas the decline of 286 mL following resection of 3–5 segments seems more likely to have a detrimental clinical impact on overall functionality and quality of life. Although this difference appears to be small, the preservation in pulmonary function associated with lesser resection may prove to be more significant for those patients with marginal pulmonary function and for those who will require additional pulmonary resections in the future for treatment of metachronous lung cancers. Similar to the findings of Keenan and colleagues [12], better preservation of DLCO was also noted in the lesser resection group. Unlike FEV_1_, it is less clear what degree of change in DLCO represents a clinically significant decline.

Although the decline in both FEV_1_ and DLCO following resection was less in the 1–2 segment cohort, only absolute (or observed) decline in FEV_1_ and DLCO reached statistical significance when comparing the two groups. Percent predicted values are derived using reference values obtained from normal or healthy patient with similar anthropometric characteristics including age, height, gender, and ethnicity. Differences in predicted values may occur when using different reference sources [22]. Many patients in our study had either preoperative or postoperative testing performed at outside institutions, where the reference sources used may have varied. The PFT’s were also performed at two different points in time, possibly affecting the reference range used based on patient age. These factors may explain the lack of statistical significance for the percent predicted difference seen in our study. The absolute change in spirometric values is likely a better representation of loss in pulmonary function following resection, as the postoperative results can be compared to each individual patient’s baseline function.

Our study does have a number of limitations, including its retrospective nature and the inherent associated selection bias. A greater proportion of patients underwent thoracotomy in the 3–5 segment cohort, which may have had a negative impact on follow-up pulmonary function. However, this effect should have been minimized at a median follow-up of 1 year. The indication for obtaining postoperative PFT’s in the current series was variable. Although most PFT’s were obtained as part of our routine postoperative screening, other indications did not preclude inclusion into the study. Patient effort is an uncontrollable variable that also likely accounted for some variation in PFT results, a problem that is inherent to effort-based testing such as PFT. Finally, quality of life measures, such as exercise testing or need for postoperative oxygen supplementation, were not included in the current study.

## Conclusion

In conclusion, parenchymal-sparing resections resulted in better preservation of postoperative pulmonary function in the present series. Functional preservation at a median of one year following resection of 1–2 anatomic segments was significantly better compared to resection of 3–5 segments for FEV_1_ and DLCO, suggesting a long-term functional benefit to parenchymal-sparing anatomic resections. The results from prospective randomized studies currently underway in Japan (JCOG0802/WJOG4607L) and North America (CALGB 140503) assessing the oncologic utility, pulmonary functional changes, and quality of life following sublobar resection versus lobectomy for stage I NSCLC will provide further insight into the most favorable approach to early stage NSCLC.
